# Reliability, clinical performance and trending ability of a pulse oximeter and pulse co-oximeter, in monitoring blood oxygenation, at two measurement sites, in immobilised white rhinoceros (*Ceratotherium simum*)

**DOI:** 10.1186/s12917-024-04179-5

**Published:** 2024-07-16

**Authors:** Thembeka K. Mtetwa, Edward P. Snelling, Peter E. Buss, Ashleigh C. Donaldson, Annette Roug, Leith C. R. Meyer

**Affiliations:** 1 Centre for Veterinary Wildlife Research, Faculty of Veterinary Science, University of Pretoria, Onderstepoort, South Africa; 2 Department of Anatomy and Physiology, Faculty of Veterinary Science, University of Pretoria, Onderstepoort, South Africa; 3 Department of Paraclinical Sciences, Faculty of Veterinary Science, University of Pretoria, Onderstepoort, South Africa; 4 Veterinary Wildlife Services, South African National Parks, Kruger National Park, Skukuza, South Africa; 5 Department of Production Animal Studies, Faculty of Veterinary Science, University of Pretoria, Onderstepoort, South Africa; 6 School of Physiology, University of the Witwatersrand Medical School, 7 York Road, Parktown, 2193 South Africa

**Keywords:** Chemical immobilisation, Oxygen-haemoglobin saturation, Wildlife

## Abstract

**Background:**

Monitoring blood oxygenation is essential in immobilised rhinoceros, which are susceptible to opioid-induced hypoxaemia. This study assessed the reliability, clinical performance and trending ability of the Nonin PalmSAT 2500 A pulse oximeter’s and the Masimo Radical-7 pulse co-oximeter’s dual-wavelength technology, with their probes placed at two measurement sites, the inner surface of the third-eyelid and the scarified ear pinna of immobilised white rhinoceroses. Eight white rhinoceros were immobilised with etorphine-based drug combinations and given butorphanol after 12 min, and oxygen after 40 min, of recumbency. The Nonin and Masimo devices, with dual-wavelength probes attached to the third-eyelid and ear recorded arterial peripheral oxygen-haemoglobin saturation (SpO_2_) at pre-determined time points, concurrently with measurements of arterial oxygen-haemoglobin saturation (SaO_2_), from drawn blood samples, by a benchtop AVOXimeter 4000 co-oximeter (reference method). Reliability of the Nonin and Masimo devices was evaluated using the Bland-Altman and the area root mean squares (ARMS) methods. Clinical performance of the devices was evaluated for their ability to accurately detect clinical hypoxemia using receiver operating characteristic (ROC) curves and measures of sensitivity, specificity, and positive and negative predictive values. Trending ability of the devices was assessed by calculating concordance rates from four-quadrant plots.

**Results:**

Only the Nonin device with transflectance probe attached to the third-eyelid provided reliable SpO_2_ measurements across the 70 to 100% saturation range (bias − 1%, precision 4%, ARMS 4%). Nonin and Masimo devices with transflectance probes attached to the third-eyelid both had high clinical performance at detecting clinical hypoxaemia [area under the ROC curves (AUC): 0.93 and 0.90, respectively]. However, the Nonin and Masimo devices with transmission probes attached to the ear were unreliable and provided only moderate clinical performance. Both Nonin and Masimo devices, at both measurement sites, had concordance rates lower than the recommended threshold of ≥ 90%, indicating poor trending ability.

**Conclusions:**

The overall assessment of reliability, clinical performance and trending ability indicate that the Nonin device with transflectance probe attached to the third-eyelid is best suited for monitoring of blood oxygenation in immobilised rhinoceros. The immobilisation procedure may have affected cardiovascular function to an extent that it limited the devices’ performance.

**Supplementary Information:**

The online version contains supplementary material available at 10.1186/s12917-024-04179-5.

## Background

Appropriate monitoring of blood oxygenation is essential in white rhinoceros (*Ceratotherium simum*) because of their susceptibility to opioid-induced hypoxaemia during chemical immobilisation [1–3]. Clinical hypoxaemia is indicated as peripheral arterial oxygen-haemoglobin saturation (SpO_2_) of ≤ 95% in dogs [4]. The exact definition of clinical hypoxaemia in rhinoceros is unknown, owing to a lack of reported consequences of low arterial oxygen-haemoglobin saturation states in this species. Since rhinoceros and horses are closely related, we assume that clinical hypoxaemia in rhinoceros is similar to that of horses. In horses that suffer from clinical disease, oxygen therapy is usually recommended when SpO_2_ is ≤ 90% [5]. Hypoventilation, typically indicated by increased arterial PCO_2_ (> 45 mmHg), is a common cause of hypoxaemia during chemical immobilisation and is exacerbated by ventilation-perfusion mismatch, impaired pulmonary gas exchange and blood shunting [3, 6–8]. Immobilised rhinoceros also develop muscle tremors and hypermetabolism, which worsens hypoxaemia by increasing oxygen utilisation and depleting limited oxygen reserves [2, 3, 7, 8]. When hypoxaemia is left untreated, it can cause organ failure, resulting in morbidity and mortality [9]. Therefore, the availability of continuous, and non-invasive methods to appropriately monitor arterial oxygen-haemoglobin saturation (SaO_2_) is crucial in the early detection of hypoxaemia, allowing for intervention before the situation deteriorates, thus reducing immobilisation-associated risks.

A traditional pulse oximeter uses dual-wavelength (660 and 940 nm) technology to measure SpO_2_ continuously and non-invasively, making it a convenient ‘go-to’ method to monitor blood oxygenation of mammals [10–13]. Recent findings from immobilised white rhinoceros show that SpO_2_ measurements from a Nonin PalmSAT 2500 A pulse oximeter are reliable when a transflectance probe is placed under the third-eyelid [14]. Conversely, pulse oximeter clip probes are attached to the scarified skin surface of an ear pinna, particularly in rhinoceros [2, 3, 7, 8, 13, 15–17], with no evidence of the reliability of this approach. Consequently, there is a need to determine the reliability of real-time SpO_2_ readings when a pulse oximeter probe is attached to a scarified ear pinna in rhinoceros, which has shown to be reliable in horses [18]. In addition, pulse oximeters’ dual-wavelength technology cannot distinguish between different haemoglobin species [19, 20], which might be limiting as the light absorption spectra of carboxyhaemoglobin and methaemoglobin are similar at the infrared spectral region (940 nm), possibly affecting the reliability of SpO_2_ measurements [19].

A potential alternative to the Nonin device may be the Masimo Radical-7 pulse co-oximeter, with an advanced signal extraction technology, which also uses non-invasive probes. Some of the Masimo probes [e.g. digital clip and specialty sensors] emit multi-wavelengths of light, between 500 and 1400 nm, to continuously measure SpO_2_, haemoglobin concentration, carboxyhaemoglobin and methaemoglobin, while other probes [e.g. low noise cabled sensors [(LNCS and LNS)] employ traditional dual-wavelength (660 and 940 nm) technology, similar to traditional pulse-oximetry, but with the advantage that the Masimo device, using either dual- or multi-wavelength probes, has a specialised feature (i.e. advanced signal extraction technology) that allows for SpO_2_ measurements even when peripheral tissue perfusion is poor and when there is excessive movement from the animal [20–24]. Studies in anaesthetised dogs [22], sheep [24], and horses [23] have compared the SpO_2_ measurements from the Masimo Radical-7 pulse co-oximeter and SaO_2_ measured by a benchtop co-oximeter. In dogs and horses, the Masimo device is reliable when the probes are placed on the tongue or lip [21–23], but in sheep, it is unreliable when probes are also placed on the tongue [24]. To date, there have been no studies using this device in rhinoceros, or any other wildlife to our knowledge.

Therefore, this study assessed the reliability of the Nonin PalmSAT 2500 A pulse oximeter’s and Masimo Radical-7 pulse co-oximeter’s dual-wavelength technology, with their probes attached to two measurement sites, the inner surface of the third-eyelid and the scarified ear pinna, in immobilised white rhinoceros. Reliability of the Nonin and Masimo devices was evaluated using Bland-Altman analysis and area root mean squares (ARMS) to compare their SpO_2_ measurements obtained from Nonin and Masimo devices to the SaO_2_ measurements obtained from the reference method, a benchtop AVOXimeter 4000 co-oximeter. We also assessed the clinical performance of the Nonin and Masimo devices for their ability to accurately detect clinical hypoxaemia (SpO_2_ ≤ 90%) by calculating sensitivities, specificities, and positive and negative predictive values and receiver operating characteristic (ROC) curves. Lastly, we assessed the trending ability of the devices using concordance rates derived from four-quadrant plots as a measure of the devices’ ability to track the magnitude and direction of change in blood oxygenation. We hypothesised that the traditional Nonin pulse oximeter and the new Masimo Radical-7 pulse co-oximeter (incorporating advanced signal extraction technology), recording SpO_2_ with dual-wavelength probes attached to the two measurement sites, could reliably estimate SaO_2_, detect hypoxaemia, and track changes in blood oxygenation in immobilised white rhinoceros.

## Methods

### Animals

A convenience sample of eight wild-caught male white rhinoceros (*Ceratotherium simum*) (four to five years-old), all originated from and inhabited in one of the South African National Parks, namely the Kruger National Park (KNP), were captured and brought to dedicated holding facilities at Veterinary Wildlife Services, Skukuza, KNP, South Africa (23° 49’ 60 S, 31° 30’ 0 E; altitude ~ 320 m). The rhinoceros were habituated to captivity for six weeks prior to the commencement of the study. Rhinoceros were fed lucerne (*Medicago sativa*) and teff hay (*Eragrostis tef*), and provided water *ad libitum*. All procedures, including the consent to collect samples, were approved by the University of Pretoria’s Animal and Research Ethics Committees (REC246-19 and REC011-21) and the South African National Park’s (custodians of the study animals) Scientific and Animal Use and Care Committee (012/20) and carried out in accordance with their guidelines and regulations.

### Study design

Data were collected opportunistically as part of a prospective, randomised, controlled crossover and non-blinded study on the broad physiological effects of opioid-based drug combinations on white rhinoceros.

### Immobilisation

**Fig. 1:**
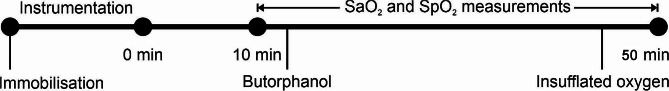
A schematic time-series of the experimental protocol showing the sequence of immobilisation, instrumentation and data collection, as well as butorphanol at 12 min and insufflated oxygen at 40 min post recumbency, before the immobilisation was reversed and the procedure ended. Each procedure lasted approximately one hour and data collection lasted for 40 min. Arterial blood samples were at taken at- 10, 15, 20, 30, 40, 45 and 50 min into heparinised syringes. Simultaneously, Nonin and Masimo SpO_2_ measurements were manually recorded from each measurement site

Each rhinoceros was subject to the following four immobilisation protocols: 1(1) etorphine (M99; Voluplex, Mnandi, Centurion, South Africa) and injectable saline, (2)^2^ etorphine and azaperone (Stresnil; Elanco, ON, Canada), (3) etorphine and midazolam (Dazonil; Wildlife Pharmaceuticals Pty Ltd, White River, South Africa), and^4^ (4) etorphine and medetomidine (Metonil; Wildlife Pharmaceuticals Pty Ltd). The protocols were delivered in a random order (www.randomiser.org), with a two-week washout period between immobilisations, and the doses calculated according to body mass (Table S1). The drug protocols were administered intramuscularly using a 3 mL plastic dart with 60 mm uncollared needle propelled by a compressed air rifle (Dan-Inject International SA, Skukuza, South Africa) aimed at the trapezius muscle in the nuchal hump area. Rhinoceros were given intravenous butorphanol (Butonil; Wildlife Pharmaceuticals Pty Ltd) at 12 min after recumbency. At 40 min, they were insufflated with oxygen intranasally (15 L/min flow rate) with the tube advanced into a single nasal passage to the level of the medial canthus of the eye, to improve blood oxygenation (see Fig. 1 for the experimental protocol).

### Benchtop co-oximetry measurements of SaO_2_

When the rhinoceros was recumbent, in a lateral position and blindfolded with its head supported by a cushion, the inner surface of the upper ear was aseptically prepared and a 25 cm, 22 Gauge over-the-needle intravenous catheter (Introcan; B Braun Medical Inc, Melsungen, Germany) was inserted into the medial auricular artery for blood sample collection. The arterial catheter line was flushed with heparinised saline (0.9% sodium chloride) and a complete washout of the saline was done prior to blood sampling. Arterial blood (3 mL) was collected anaerobically at 10, 15, 20, 30, and 40 min after recumbency into 3 mL syringes (spray-dried, calcium-balanced lithium heparin, final concentration of 50 IU/mL; BD Medical, NJ, USA). During oxygen insufflation, additional arterial blood samples were collected at 45 and 50 min. In addition, due to technical issues with data collection in other studies undertaken concurrently with the present study, several immobilisation protocols had to be redone, allowing for additional sampling for this study.

All arterial blood samples were immediately placed on ice and before analysis the syringes containing the blood were rolled gently to ensure homogeneity. Arterial oxygen-haemoglobin saturation (SaO_2_; %) was measured from the blood samples, within 15 min of collection, using the benchtop AVOXimeter 4000 co-oximeter with test cuvettes (Surgical Innovations, Johannesburg, South Africa). An optical quality control of the co-oximeter was performed daily using yellow and orange optical filters to calibrate and verify that the optics used for measurements were not obscured by residual blood debris. The benchtop co-oximeter uses specific multi-wavelengths of light (488.4, 520.1, 562.4, 585.2, 597.5, 621.7 and 671.7 nm) to measure SaO_2_, carboxyhaemoglobin and methaemoglobin (AVOXimeter 4000 user manual). The SaO_2_ values from the co-oximeter were used as reference measurements and compared against the simultaneous SpO_2_ values from the Nonin and Masimo devices. Even though human benchtop co-oximeters have not been validated in rhinoceros, they have been used successfully to validate the use of pulse oximetry in anaesthetised impala [25] and horses [23, 26]. Furthermore, the human benchtop co-oximeters’ algorithms are likely suitable for rhinoceros because the spectrophotometric characteristic of haemoglobin (i.e., infrared absorbance of oxyhaemoglobin and deoxyhaemoglobin) are similar among human, horses and white rhinoceros [17]. Carboxyhaemoglobin and methaemoglobin concentrations were 1 ± 0.2% and 1 ± 0.5% (mean ± SD; %), respectively, in the present study. Therefore, these haemoglobin derivatives did not influence oxygen saturation measurements.

### Pulse oximetry and pulse co-oximetry measurements of SpO_2_

**Fig. 2:**
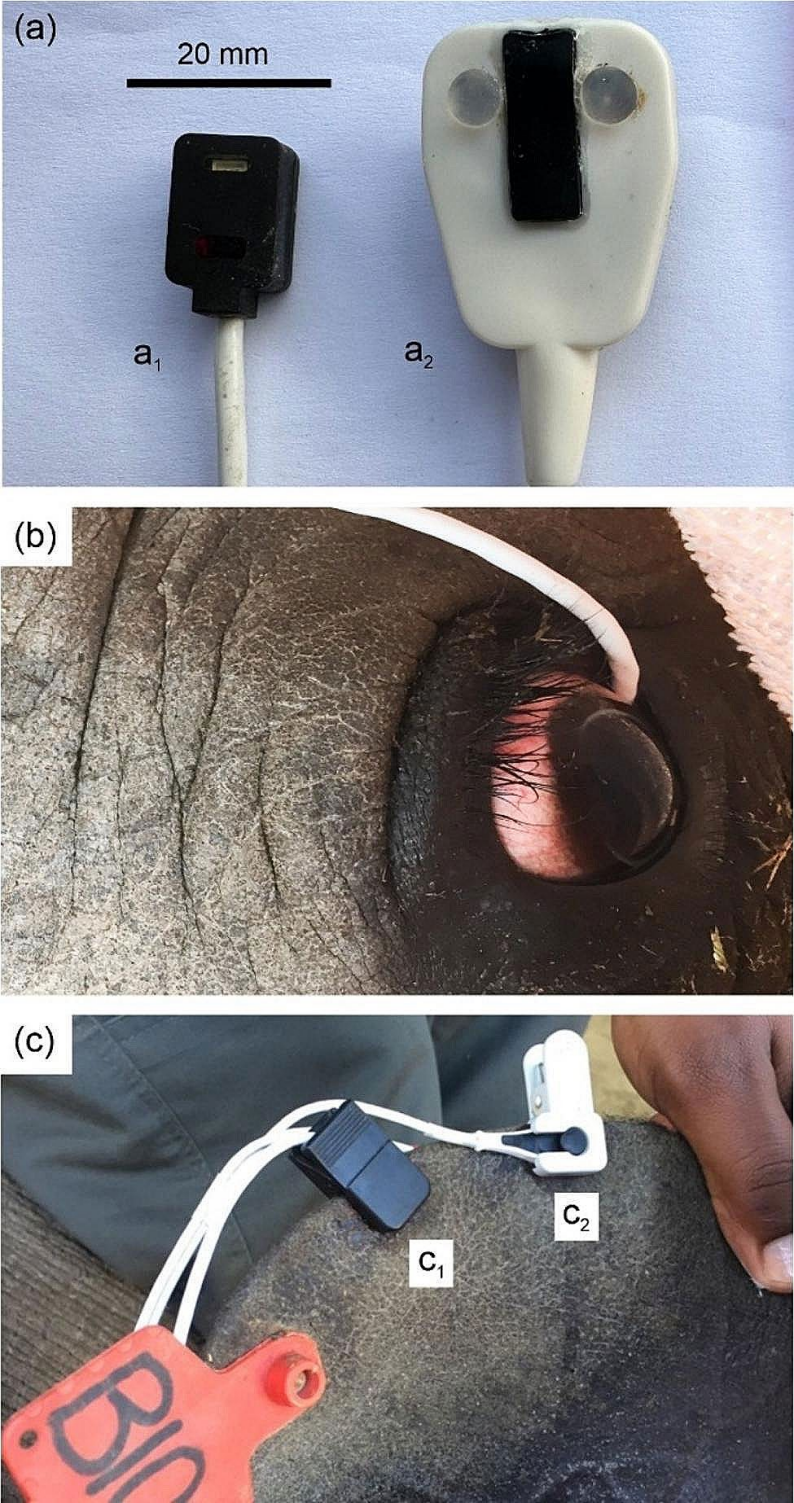
The Nonin 2000T transflectance probe [Fig. 2 (a_1_); attached to the Nonin PalmSAT 2500 A pulse oximeter] and the Masimo LNCS TF-I AH transflectance probe [Fig. 2 (a_2_); attached to the Masimo Radical-7 pulse co-oximeter] were placed on the third-eyelid (nictitating membrane mucosa) of white rhinoceros (*Ceratotherium simum*) [Fig. 2 (b)]. The Nonin probe was attached before the animal was placed in a recumbent position (either on the left or right side), so that it was always on the third-eyelid of the side that the rhinoceros rested (i.e., lower eye). The Masimo probe was placed on the upper-facing third-eyelid (i.e., upper eye) so that its position could be checked more regularly. In addition, a Nonin 2000SL lingual transmission clip probe [Fig. 2 (c_1_); attached to the Nonin device] and a Masimo LNS YI AH SpO_2_ animal multisite transmission clip probe [Fig. 2 (c_2_); attached to the Masimo device], were placed adjacent each other on the upper ear, on the border of the ear pinna (upper helix), after carefully scarifying the skin on both sides with a scalpel blade. Once all probes were positioned, the rhinoceros was blindfolded before commencing pulse oximeter and pulse co-oximeter measurements

A Nonin PalmSAT 2500 A pulse oximeter with a 2000T transflectance probe (Nonin Medical Inc, North Plymouth, USA) [Fig. 2: (a_1_)] and a Masimo Radical-7 pulse co-oximeter with a LNCS TF-I AH transflectance probe (Masimo Corporation., Irvine, USA) [Fig. 2: (a_2_)] were cleaned with alcohol, covered in lubricant gel (K-Y jelly) and placed under the third-eyelid (nictitating membrane mucosa) of each eye [Fig. 2: (b)]. The Nonin transflectance probe was inserted prior to the animal being placed in a lateral position (either on the left or right side), so that it was always attached to the third-eyelid of the lowermost eye on the side the rhinoceros rested. The Masimo transflectance probe was placed on the third-eyelid of the uppermost eye so that it could be more closely monitored. The transflectance probes fitted comfortably into the loose space between the sclera and the third-eyelid. The probes’ relatively small sizes in comparison to the rhinoceros eye, and together with their smooth and rounded edges [see Fig. 2: (a) and (b)], ensured that no visible physical damage occurred. The eyes of the rhinoceros remain open and fixed during immobilisations, so the palpebral and corneal reflexes are not relied on to determine the depth of immobilisation. Furthermore, the presence of the probes did not affect the assessment of immobilisation depth.

Once an arterial catheter line was secured, a 2000SL lingual transmission probe [Fig. 2: (c_1_)] connected to a second Nonin device, and a LNS YI AH SpO_2_ transmission probe [Fig. 2: (c_2_)] connected to a second Masimo device, were attached to the upper ear pinna. The probes were placed adjacent to each other on the same ear, at the border of the ear pinna (upper helix), after carefully scarifying and removing the pigmented epidermal layer of the skin by scraping both sides with a scalpel blade until superficial bleeding was noticed and ensuring that the probe placement was standardised for all study animals.

Peripheral arterial oxygen-haemoglobin saturation (SpO_2_; %), pulse rate and pulse quality were recorded in sets of three (i.e., triplicate), from the Nonin and Masimo devices. Signal pulse quality was indicated by a coloured LED built into the Nonin devices, where green signifies good, amber intermediate and red poor. Signal pulse quality from the Masimo devices was indicated by the signal identification and quality indicator (signal IQ) that corresponds to the peak of an arterial pulsation. The SpO_2_ and pulse rate readings on the Masimo devices were taken only when the vertical line (positive deflection) of the signal IQ was consistently at its highest peak, indicating that the measurements displayed were of a good signal pulse quality. Sensitivity mode on the Masimo devices was set at “adaptive probe off detection”, which is recommended when movement at the measurement site may cause erroneous readings. Readings from the Nonin and Masimo devices were recorded simultaneously or within 30 s of collecting arterial blood samples (see above). The readings were all at set averaging times of 10 s and recorded in the following order: (1) Nonin third-eyelid, (2) Masimo third-eyelid, (3) Nonin ear, and (4) Masimo ear.

### Data handling

Means and standard deviations (SD) were calculated for the triplicate SpO_2_ measurements at each time point and exclusion criteria [14, 25] were applied to exclude poor signal pulse quality SpO_2_ readings from Nonin devices. All recordings from the Masimo devices were of good signal pulse quality (i.e., readings were taken only when there was a consistent positive deflection of the vertical line of the signal IQ). Therefore, the acceptable “pass data” from the Masimo devices comprised only SpO_2_ measurements where the SD between the triplicate SpO_2_ measurements was < 3%, which indicated stable and consistent measurements, whereas “excluded data” comprised SpO_2_ measurements where the SD was ≥ 3% [14, 25].

### Statistical analyses

Reliability (i.e., accuracy and precision) of the Nonin and Masimo SpO_2_ measurements, at each measurement site, was determined using Bland-Altman method for multiple observations [27] and the “pass data” SpO_2_ measurements were compared with SaO_2_ measurements. The Bland-Altman method determined the bias (accuracy), precision of the measurements (SD), and the limits of agreement (LOA) between the paired measurements (bias ± 1.96 × SD). Bias is the average mean difference (i.e., SpO_2_ – SaO_2_), and precision is a measure of the variability (SD) of the paired measurements. For Bland-Altman method analysis to meet 90% power at an α of 0.05 and β of 0.10, a minimum of 17 paired data (SpO_2_-SaO_2_) were required. Area root mean squares (ARMS) was calculated to determine the overall reliability [27] by combining the accuracy and precision using the equation:

$$\:\text{A}\text{R}\text{M}\text{S}=\:\sqrt{\frac{\sum\:_{i=n}^{n}{({\text{S}\text{p}\text{O}}_{2}-{\text{S}\text{a}\text{O}}_{2})}^{2}}{n}}$$

Where *n* is the sample size of the paired measurements. Guidelines by the FDA and ISO [28, 29] have certified pulse oximetry as reliable when the comparisons with co-oximetry are ≤±3% for bias, ≤3% for precision and ≤4% for ARMS [27].

Clinical performance of the Nonin and Masimo devices was also assessed by calculating the devices’ ability to detect clinical hypoxaemia at each measurement site. Calculations were made of sensitivities, specificities, positive and negative predictive values and receiver operating characteristic (ROC) curves, which are performance indicators of the devices’ ability to detect normoxia and clinical hypoxaemia. Clinical hypoxaemia has not yet been defined clearly for rhinoceros owing to a lack of reported consequences of low arterial oxygen-haemoglobin saturation states. Therefore, in this study, we used SpO_2_ ≤ 90% as a threshold for clinical hypoxaemia as it has previously been recommended as a threshold at which oxygen therapy is required for horses (not as large but closely related to rhinoceros) treated for pulmonary disease [5]. A lower cut-off value for detection of severe hypoxaemia (i.e., SpO_2_ ≤ 80%) was also evaluated. Sensitivity is the percentage of hypoxaemic rhinoceros correctly identified as hypoxaemic by the Nonin and Masimo devices (i.e., number of true positives divided by the sum of true positives and false negatives). Specificity is the percentage of normoxic rhinoceros correctly identified as clinically normal (i.e., number of true negatives divided by the sum of true negatives and false positives). A positive predictive value is the likelihood that a rhinoceros identified as hypoxaemic is actually hypoxaemic (i.e., number of true positives divided by the sum of true positives and false positives). A negative predictive value is the likelihood that a rhinoceros identified as normoxic is actually clinically normal (i.e., number of true negatives divided by the sum of true negatives and false negatives). The ROC curve shows the relationship between sensitivity (%) and 100 (%) – specificity (%) [30]. One hundred minus specificity [100 (%) – specificity (%)] is the false positive ratio which gives the percentage of wrongly identified hypoxaemic rhinoceros (i.e., number of false positives divided by the sum of false positives and true negatives). Area under the curves (AUC) were derived from the ROC curves for both Nonin and Masimo devices, at the two measurement sites and used to determine the clinical performance of the devices in detecting clinical hypoxaemia using the following guidelines: 0.50 < AUC ≤ 0.70 is low, 0.70 < AUC ≤ 0.90 moderate and 0.90 < AUC ≤ 1.00 high performance [31].

Trending ability of SpO_2_ measured by the Nonin and Masimo devices, at both measurement sites, was assessed by tracking the devices’ ability to follow SaO_2_ measured by the reference method, the benchtop AVOXimeter 4000 co-oximeter. Two sequential oxygen saturation values (i.e., ΔSaO_2_ and ΔSpO_2_) were used to plot four-quadrant plots. A central exclusion zone of ± 3% was used to exclude paired data points with minimal difference. Concordance rates, defined as the percentage of ΔSaO_2_ and ΔSpO_2_ paired data points that lie within the two quadrants of trending agreement (i.e., lower left and upper right), were calculated outside of the central exclusion zones of the four-quadrant plots. According to Critchley and co-workers, concordance rates of ≥ 90% are considered acceptable for assessing trending ability [32].

Statistical analyses were performed using GraphPad Prism version 9.3.1 (GraphPad Software, La Jolla, CA, USA) and R 4.4.0 statistical software (R Core Team, 2020; R Foundation for Statistical Computing, Austria; https://www.R-project.org). *P* < 0.05 was considered statistically significant.

## Results

### Reliability of the Nonin and Masimo devices at the two measurement sites

More than 80% of the recorded Nonin PalmSAT 2500 A pulse oximeter and Masimo Radical-7 pulse co-oximeter SpO_2_ data (i.e., % “pass data”), at the two measurement sites, met the criteria for further analysis (Table 1).

**Table 1:** Number of time-matched (paired) measurements of peripheral arterial oxygen-haemoglobin saturation (SpO_2_) from the Nonin PalmSAT pulse oximeters and Masimo Radical-7 pulse co-oximeters at the two measurement sites (third-eyelid and ear), compared with arterial oxygen-haemoglobin saturation (SaO_2_) measured from the reference method, a benchtop AVOXimeter 4000 co-oximeter, in eight immobilised white rhinoceros (*Ceratotherium simum*). Presented are the total number of paired measurements for all the data before the exclusion criteria was applied (“all data”), the Nonin PalmSAT pulse oximeters signal pulse quality (green, amber, or red), total number of paired measurements after exclusion criteria were applied (“pass data”), the total number of paired measurements excluded (“excluded data”), as well as the exclusion criteria applied. SD, standard deviation. Sample size (*n*)

Device measurement site	All data (*n*)	Pulse signal quality indicator light: % of total readings	Excluded data (*n*) and exclusion criteria		Pass data (*n*)
			Triplicate (i.e., three) SpO_2_ with SD > 3%	Poor pulse signal quality (i.e., red)	
Nonin third-eyelid	283	Green: 76.0% Amber: 23.7% Red: 0.3%	16 (5.7%)	1 (0.3%)	266 (94.0%)^*^
Masimo third-eyelid	291		39 (13.0%)	N/A	252 (87.0%)^†^
Nonin ear	255	Green: 74.0% Amber: 19.0% Red: 7.0%	26 (10.0%)	21 (8.0%)	208 (82.0%)^*^
Masimo ear					
	251		43 (17.0%)	N/A	208 (83.0%)^†^

^*^For the Nonin PalmSAT devices, “pass data” comprised of SpO_2_ measurements where the signal pulse quality was indicated by a green or amber light (good and intermediate signal pulse quality) and the SD between the triplicate SpO_2_ measurements was < 3%. ^†^For the Masimo Radical-7 devices, “pass data” comprised of triplicate SpO_2_ measurements with SD < 3% (good signal pulse quality was not used as exclusion criteria because all readings were taken only when the device indicated a consistent positive deflection the vertical line of the signal IQ)

**Fig. 3:**
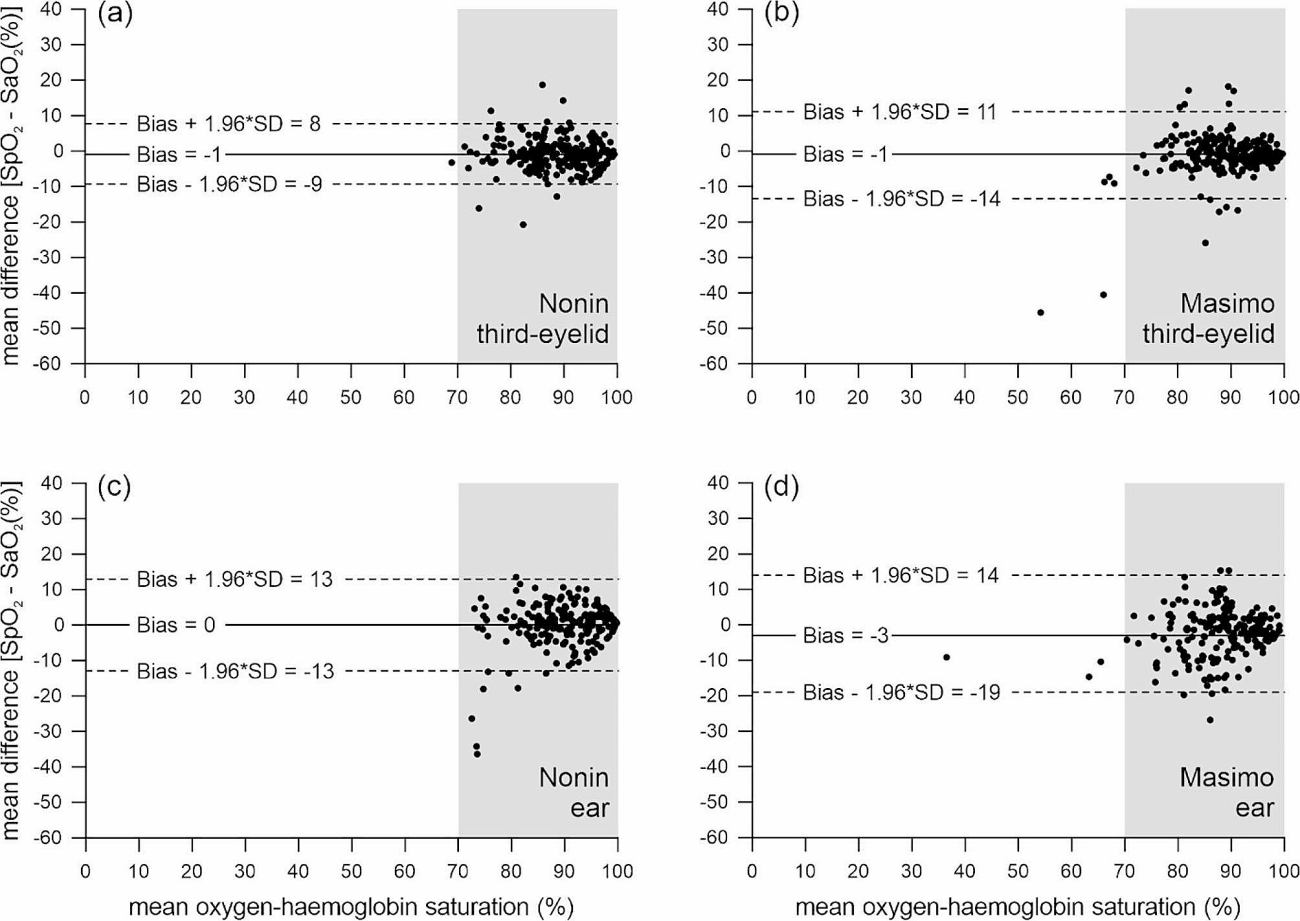
Bland-Altman plots, showing the level of agreement as a measure of reliability of arterial peripheral arterial oxygen-haemoglobin saturation (SpO_2_) measured by the Nonin and Masimo devices with probes attached at four different measurement sites when compared with arterial oxygen-haemoglobin saturation (SaO_2_) measured by a reference method, benchtop AVOXimeter 4000 co-oximeter, in eight immobilised white rhinoceroses. Figure 3 (**a**) and (**b**) assesses reliability of the Nonin and Masimo devices when the transflectance probes were placed against the third-eyelids (*n* = 226 and 230, respectively), and Fig. 3 (**c**) and (**d**) when the Nonin and Masimo transmission probes attached to the ear (*n* = 196 and 193, respectively). The percent mean difference between SpO_2_ and SaO_2_ is plotted against the percent mean oxygen-haemoglobin saturation measurements (i.e., SpO_2_ and SaO_2_). The grey shaded regions represent the Bland-Altman analysis at the manufacturer’s claimed performance range of 70–100%. The estimated bias is represented by the solid line, and limits of agreement [bias ± 1.96$\:*$standard deviation (SD)] are represented by the dashed lines.

The Nonin device with a transflectance probe placed under the third-eyelid, compared to the benchtop co-oximeter, provided 266 paired SpO_2_-SaO_2_ measurements for comparisons. Mean SpO_2_ measurements recorded from this device, at this measurement site, ranged from 34 to 100% and the corresponding SaO_2_ measurements ranged from 12 to 100%. At the manufacturer’s claimed performance range of 70 to 100%, SpO_2_ measurements were accurate, and although imprecise, had an acceptable overall reliability (bias − 1%, precision 4% and ARMS 4%) (Fig. 3; Table 2). Across the entire 0 to 100% saturation range, SpO_2_ measurements were accurate, but imprecise, and thus overall unreliable (bias 1%, precision 6% and ARMS 6%). Below 70%, SpO_2_ measurements were inaccurate, imprecise, and thus overall unreliable (bias 8%, precision 10% and ARMS 16%).

**Table 2:** Bland-Altman analysis and area root mean squares (ARMS) results as a measure of reliability of the Nonin PalmSAT 2500 A pulse oximeters (SpO_2_) and Masimo Radical-7 pulse co-oximeters (SpO_2_) at two measurement sites (third-eyelid and ear) compared with a reference method, a benchtop AVOXimeter 4000 co-oximeter, in eight immobilised white rhinoceros (*Ceratotherium simum*) after exclusion criteria were applied (i.e., represents only “pass data”). SpO_2_, peripheral oxygen-haemoglobin saturation. SaO_2_, arterial oxygen-haemoglobin saturation. LOA, limits of agreement (minimum to maximum). *n* represents the sample size of the pass data. Bold denotes results at the manufacturer’s claimed performance range of 70 to 100%.

Device measurement site	Ranges (%)	Sample size (*n*)	Bias (%)	Precision (%)	ARMS (%)	LOA
Nonin third-eyelid	0–100	266	1*	6	6	-12 to 13
	**70**–**100**	**226**	-1*	**4**	4*	**-9 to 8**
	< 70	40	8	10	16	-11 to 28
	70–79	25	2*	5	6	-8 to 13
	80–89	92	0*	4	4*	-8 to 8
	90–100	109	-2*	4	4*	-10 to 6
Masimo third-eyelid	0–100	252	0*	9	8	-17 to 16
	**70**–**100**	**230**	-1*	**6**	**6**	**-14 to 11**
	< 70	23	8	19	20	-29 to 45
	70–79	24	-1*	11	11	-23 to 22
	80–89	91	0*	6	6	-12 to 12
	90–100	114	-2*	4	5	-11 to 6
Nonin ear	0–100	208	1*	7	7	-13 to 14
	**70**–**100**	**196**	0*	**7**	**7**	**-13 to 13**
	< 70	12	10	8	13	-5 to 26
	70–79	20	4	4	6	-4 to 13
	80–89	76	1*	6	6	-11 to 13
	90–100	100	-2*	7	7	-15 to 12
Masimo ear	0–100	208	-1*	11	11	-22 to 19
	**70**–**100**	**193**	-3*	**8**	**9**	**-19 to 14**
	< 70	15	13	21	24	-28 to 55
	70–79	19	1*	7	7	-13 to 15
	80–89	73	0*	7	7	-14 to 13
	90–100	101	-5	9	10	-23 to 12

*Results considered acceptable for the Nonin PalmSAT 2500 A pulse oximeters (bias ≤ ± 3%, precision ≤ 3%, ARMS ≤ 4%) and Masimo Radical-7 pulse co-oximeter (bias ≤ ± 3%, precision ≤ 3%, ARMS ≤ 3%) according to manufacturer guidelines (Nonin Medical Inc., Minnesota, USA and Masimo Corp., Irvine, California, respectively), the United States Food and Drug Administration (FDA) and the International Organization for Standardization (DIN EN ISO 80601-2-61)

The Masimo device with a transflectance probe placed under the third-eyelid, compared to the benchtop co-oximeter, provided 252 paired SpO_2_-SaO_2_ measurements. Mean SpO_2_ ranged from 19 to 99% and SaO_2_ from 30 to 100%. The SpO_2_ measurements were accurate but imprecise and thus unreliable within the 70 to 100% (bias − 1% precision 6% and ARMS 6%) (Fig. 3) and across the entire 0 to 100% saturation range (bias − 1% precision 9% and ARMS 8%) (Table 2). Below 70%, SpO_2_ measurements were inaccurate, imprecise, and thus unreliable (bias 8%, precision 19% and ARMS 20%).

The Nonin device with a transmission probe placed on the ear, compared to the benchtop co-oximeter, allowed for 208 paired SpO_2_-SaO_2_ measurements. Mean SpO_2_ ranged from 55 to 100% and SaO_2_ from 35 to 100%. The SpO_2_ measurements were accurate but imprecise and thus overall unreliable within 70 to 100% (bias 0%, precision 7% and ARMS 7%) (Fig. 3) and across the entire 0 to 100% saturation range (bias 1%, precision 7% and ARMS 7%) (Table 2). Below 70%, SpO_2_ measurements were inaccurate, imprecise, and thus unreliable (bias 10%, precision 8% and ARMS 13%).

The Masimo device with a transmission probe placed on the ear, compared to the benchtop co-oximeter, allowed for 208 paired SpO_2_-SaO_2_ measurements. Mean SpO_2_ ranged from 21 to 100% and SaO_2_ from 26 to 100%. The SpO_2_ measurements were accurate but imprecise and thus unreliable within 70 to 100% (bias − 3%, precision 8% and ARMS 9%) (Fig. 3) and across the entire 0 to 100% saturation ranges (bias − 1%, precision 11% and ARMS 11%) (Table 2). Below 70%, SpO_2_ measurements were inaccurate, imprecise, and thus unreliable (bias 13%, precision 21% and ARMS 24%).

### Clinical performance of the Nonin and Masimo devices at the two measurement sites

**Fig. 4:**
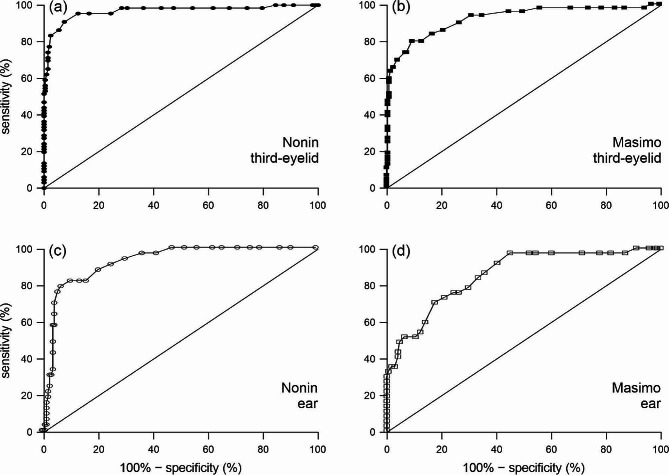
Receiver operating characteristic (ROC) curves as a measure of clinical performance of the Nonin pulse oximeter’s and Masimo pulse co-oximeter’s ability to detect clinical hypoxaemia (SpO_2_ ≤ 90%) in immobilised white rhinoceros. Figure 4 (**a**) and (**b**) assesses the clinical performance of the Nonin and Masimo devices when the transflectance probes were placed against the third eyelids and Fig. 4 (**c**) and (**d**) when the transmission probes placed on the ear. The sensitivity (%) is plotted against the 100% – specificity (%) (or false positive ratio). The line of identity indicates random clinical performance level. Results with a more severe hypoxaemia set at SpO_2_ ≤ 80% are represented in Table S2

For the detection of clinical hypoxaemia (SpO_2_ ≤ 90%), the Nonin device, with probe placed under the third-eyelid, had 90% sensitivity, 74% specificity, 84% and 83% positive and negative predictive values, respectively, with high clinical performance at detecting clinical hypoxaemia [AUC 0.93 ± 0.03 (± 95% CI)]. The Masimo device, at the same measurement site, had 94% sensitivity, 77% specificity, 85% and 91% positive and negative predictive values, respectively, with high clinical performance at detecting clinical hypoxaemia (AUC 0.90 ± 0.04). The Nonin and Masimo devices, with probes placed on the ear, had 81% and 83% sensitivities, 74% and 63% specificities, 77% and 74% positive and 78% and 75% negative predictive values, respectively, with only a moderate performance (AUC 0.86 ± 0.05 and AUC 0.79 ± 0.06, respectively) at detecting clinical hypoxaemia [Fig. 4; Table 3]. Compared to clinical hypoxaemia (SpO_2_ ≤ 90%) at the lower cut-off value for detecting severe hypoxaemia (SpO_2_ ≤ 80%), sensitivities were lower and specificities higher for both devices (Table S2).

**Table 3:** Sensitivity, specificity, positive and negative predictive values and area under the curve (AUC) values as a measure of clinical performance of the Nonin PalmSAT 2500 A pulse oximeters’ and Masimo Radical-7 pulse co-oximeters at two measurement sites (third-eyelid and ear) for the detection of clinical hypoxaemia [i.e., defined as peripheral arterial oxygen-haemoglobin saturation (SpO_2_) ≤ 90% in this present study] in eight immobilised white rhinoceros (*Ceratotherium simum*). CI, confidence intervals. Results at SpO_2_ ≤ 80% are represented in Table S2

Device measurement site	Cut-off value (i.e., clinical decision limit)	Sensitivity (95% CI) (%)	Specificity (95% CI) (%)	Positive predictive value (95% CI) (%)	Negative predictive value (95% CI) (%)	AUC* ± 95% CI	*P* value
Nonin third-eyelid	SpO_2_ ≤ 90%	90 (84 to 94)	74 (64 to 82)	84 (78 to 89)	83 (74 to 89)	0.93 **±** 0.03^§§^	< 0.0001
Masimo third-eyelid		94 (89 to 97)	77 (68 to 84)	85 (78 to 89)	91 (83 to 95)	0.90 ± 0.04^§§^	< 0.0001
Nonin ear		81 (73 to 87)	74 (65 to 82)	77 (71 to 86)	78 (66 to 84)	0.86 ± 0.05^††^	< 0.0001
Masimo ear		83 (75 to 88)	63 (53 to 73)	74 (65 to 80)	75 (64 to 83)	0.79 ± 0.06^††^	< 0.0001

^*^ Area under the ROC curves (AUC) were used to determine the Nonin and Masimo device’s clinical performance in detecting clinical and severe hypoxaemia and the following guidelines have been proposed: low 0.50 < AUC ≤ 0.70**, moderate 0.70 < AUC ≤ 0.90^††^ and high 0.90 < AUC ≤ 1.00^§§^ clinical performance [30]

### Trending ability of the Nonin and Masimo devices at the two measurement sites

**Fig. 5:**
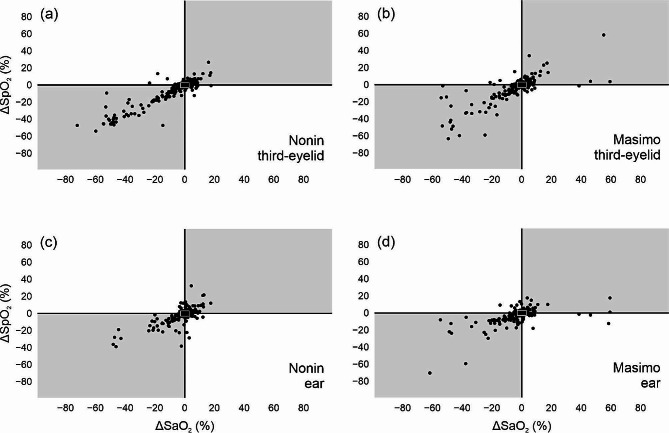
Four-quadrant plots as a measure of trending ability of the percentage change in sequential peripheral oxygen-haemoglobin saturation measurements (Δ SpO_2_) measured by the Nonin pulse oximeters and Masimo pulse co-oximeters (*y-axes*) to follow the percentage change in sequential arterial oxygen-haemoglobin saturation measurements (Δ SaO_2_), measured by the reference method, benchtop AVOXimeter 4000 co-oximeter (*x-axes*). Figure 5 (**a**) and (**b**) assesses trending ability of the Nonin and Masimo devices when the transflectance probes were placed against the third eyelids and Fig. 5 (**c**) and (**d**) when the transmission probes placed on the ear. The small white rectangles represent the central exclusion zones of ± 3%, set to exclude two sequential measurements of oxygen saturation with minimal difference. The concordance rate, defined as the percentage of paired data sets which lie within the quadrants of agreement (grey-shaded quadrants) are 82% for Nonin third-eyelid, 78% for Masimo third-eyelid, 82% Nonin ear and 76% Masimo ear

Trending ability of the Nonin and Masimo devices, at both measurement sites, was assessed using four-quadrant plots (Fig. 5). About 35% paired data points were within the central exclusion zone of ± 3% for the Nonin and Masimo devices, at both measurement sites (Fig. 5), and were not included for the calculation of concordance rates. The analysis of data outside the central exclusion zone showed poor trending ability with concordance rates below the recommended threshold of ≥ 90% for both devices at both measurement sites (82% for Nonin third-eyelid, 78% for Masimo third-eyelid, 82% for Nonin ear and 76% for Masimo ear).

## Discussion

This study showed that the Nonin PalmSAT 2500 A pulse oximeter with a 2000T transflectance probe placed under the third-eyelid provided reliable SpO_2_ measurements at the manufacturer’s claimed performance range of 70 to 100% in immobilised white rhinoceros. However, the same device with a transmission probe placed on the ear pinna, and the Masimo Radical-7 pulse co-oximeter with transflectance and transmission probes placed under the third-eyelid and ear pinna, respectively, provided unreliable SpO_2_ measurements, with noticeably poor precision across the same 70 to 100% saturation range. Both the Nonin and Masimo devices, with transflectance probes placed under the third-eyelid had high clinical performance and were generally sensitive at detecting clinically serious hypoxaemia (SpO_2_ ≤ 90%). However, the Nonin and Masimo devices, with their transmission probes placed on the ear had only moderate clinical performance at detecting clinical hypoxaemia. Overall, both the Nonin and Masimo devices, at the two measurement sites, had poor trending ability with concordance rates below the threshold of ≤ 90%, although the Nonin device fared equal best at 82%. Therefore, for overall assessment of reliability, clinical performance and trending ability, the Nonin device with its transflectance probe placed under the third-eyelid is best suited for monitoring blood oxygenation in immobilised white rhinoceros.

Reliability of pulse oximetry varies depending on the probe measurement site [11, 12, 25, 33]. Pulse oximetry studies showed that the tail in impala [25], and third-eyelid in earlier analysis in rhinoceros [14], are reliable measurement sites. Similarly, this study corroborates that the third-eyelid is a reliable measurement site for pulse oximetry in immobilised white rhinoceros. In the field, a traditional pulse oximeter probe is commonly attached on the scarified ear pinna of the rhinoceros with no knowledge of how reliable this measurement site is in this species [7, 13, 34, 35]. In this study, the results show that the scarified ear is unreliable, with SpO_2_ measurements that are generally accurate but imprecise for both Nonin and Masimo devices. The skin on the ear pinna of rhinoceros is thick and pigmented; therefore, it is common to scarify the skin by scraping it with a blade to obtain SpO_2_ readings [13, 34, 35]. However, skin scarification results in bleeding, which exposes the blood to air and thus potentially influences the SpO_2_ readings from the probes placed in the ear, which possibly contributed to the poor precision in this study.

Pulse oximetry readings may be affected by the manufacturer and model of the pulse oximeter. In this study, the Nonin device performed better than the Masimo device. Although the Masimo device generally gave accurate readings at both measurement sites [third-eyelid (bias − 1%) and ear (bias − 3%)], these readings were overall unreliable due to poor precision [third-eyelid (precision 6%) and ear (precision 8%)]. These findings are similar to those reported in anaesthetised sheep when a Masimo transmission probe was attached to the tongue (bias − 2%, precision 6%) [24]. These findings are surprising because the Masimo devices use a signal extraction technology that is not found in traditional Nonin devices [18, 23]. The signal extraction technology applies adaptive filters that separate the true arterial signal from signals caused by different physiologic artefacts (e.g. motion or light artefacts) and separates them out by evaluating the whole signal and breaking it down into its fundamental components, which is believed to improve measurement accuracy [24, 36]. Our findings show that this new signal extraction technology did not improve the reliability of the Masimo SpO_2_ measurements in our rhinoceros. Therefore, the unreliability of the Masimo device, when the transflectance probe was placed on the third-eyelid, a measurement site otherwise shown to be reliable in rhinoceros [14], could be due to the probe size and shape. Compared to the Nonin transflectance probe, the Masimo probe is larger [see Fig. 1(a_1_) vs. (a_2_)] which made it more challenging to fit securely alongside the animal’s eye, thereby potentially creating poor contact with the third-eyelid. The Masimo probe size prompted its placement on the uppermost third-eyelid to ensure close inspection and regular repositioning. Nonetheless, the Masimo probe on the third-eyelid was still unreliable when compared to the Nonin probe on the lowermost third-eyelid.

In this study, the transmission probes attached to the ear pinna using both Nonin and Masimo devices provided accurate (low bias) but overall unreliable SpO_2_ measurements. Giguére and colleagues [12] reported similar findings showing unreliability of the SpO_2_ readings from the ear, using dual-wavelength pulse oximeter transmission probes in anaesthetised foals. Additional reasons to the unreliability of the transmission probes used in our study include poor tissue perfusion and tissue thickness which inhibit accurate transmission of light through the tissue and limit its detection by the photodiode located on the opposite side of the probe.

In addition to the reliability, the clinical performance of blood oxygen measuring devices in detecting hypoxaemia is also an important consideration when assessing these devices and their measurement sites, especially during rhinoceros immobilisation, to inform proper decision making, such as when to give supplementary oxygen to hypoxaemic animals [7, 17]. Therefore, we evaluated the devices’ clinical performance in alerting clinicians of clinical hypoxaemia (i.e., SpO_2_ ≤90%), regardless of the measurement accuracy (i.e., reliability) of SpO_2_ when compared to SaO_2_. Overall, the Nonin and Masimo devices, with probes under the third-eyelid, provided acceptable detection accuracy (i.e., high clinical performance) and 92% of the cases on average, were correctly identified as clinical hypoxaemia (sensitivity of 90% and 94%, respectively), and for the rhinoceros that were correctly identified as clinical hypoxaemic, 85% on average were indeed hypoxaemic (positive predictive values of 84% and 85%, respectively). Overall, both the Nonin and Masimo devices, at both measurement sites, correctly identified normoxia. Therefore, the Nonin and Masimo devices, at both measurement sites, can detect clinical hypoxaemia in immobilised white rhinoceros. Nonetheless, in immobilised rhinoceros with a high risk of opioid-induced hypoxaemia, high sensitivities and positive predictive values are more informative than high specificities and negative predictive values, because it is important to correctly identify clinical hypoxaemia to prevent tissue hypoxia, organ failure and potential morbidity and mortality outcomes.

In addition to assessing reliability and clinical performance, it is important to also assess the devices’ ability to track both the magnitude and directional changes in blood oxygenation. The concordance rate, derived from four-quadrant plots, and used to assess trending ability, were on average 80%, which are below the acceptable threshold of ≥ 90% according to Critchley and colleagues [32] and thus indicate poor trending ability. Nonetheless, the Nonin device with probe placed under the third eyelid fared equal best (82%) even though it failed to meet the threshold of acceptable trending ability. Nonetheless, the immobilisation procedure may have affected cardiovascular function (e.g., peripheral perfusion, heart rate, and, or, oxygen saturation) to such an extent that it limited the overall performance of the devices.

## Conclusion

We have shown that the Nonin PalmSAT 2500 A pulse oximeter with a 2000T transflectance probe placed under the third-eyelid was the only device and probe combination that provided reliable SpO_2_ measurements across the manufacturer’s claimed performance range of 70 to 100%. We also showed that the Nonin and Masimo devices with probes attached under the third-eyelid provided high clinical performance at detecting hypoxaemia. The Nonin and Masimo devices and probes attached on the ear were unreliable and only provided moderate clinical performance. The Nonin and Masimo devices, at both measurement sites provided poor trending ability for monitoring blood oxygenation. Clinicians and scientists should select pulse oximeters or pulse co-oximeters, and their probe measurement sites carefully and in accordance with their needs and objectives. These decisions are important because some devices can provide inaccurate and unreliable measurements with poor trending ability when monitoring blood oxygenation, but can still be clinically acceptable for detecting clinical hypoxaemia. With overall assessment of reliability, clinical performance and trending ability, the results of this study indicate that the Nonin device, with its transflectance probe placed under the third-eyelid, is best suited for monitoring blood oxygenation in immobilised white rhinoceros.

### Electronic supplementary material

Below is the link to the electronic supplementary material.

Supplementary Material 1

## Data Availability

The datasets generated and/or analysed during the current study are available in the University of Pretoria data repository, https://figshare.com/s/56087e8a1ba4fb6c1b51.
