# Supplementary material for: Reliability, clinical performance and trending ability of a pulse oximeter and pulse co-oximeter, in monitoring blood oxygenation, at two measurement sites, in immobilised white rhinoceros (Ceratotherium simum)
Source: BMC Vet Res. 2024 Jul 16;20:319. doi: 10.1186/s12917-024-04179-5 (PMC11251122; doi:10.1186/s12917-024-04179-5)
Supplement: Supplementary file 1 — Supplementary Material 1 [file 12917_2024_4179_MOESM1_ESM.docx]

Supplementary material

Table S1 Standard drug doses based on body mass range in eight white rhinoceros (*Ceratotherium simum*)*.* Animals were weighed intermittently during the four-to-six-week habituation period and at the end of each immobilisation and reversal procedure, by encouraging them to enter a tarred crate suspended from a crane balance. Mean ± standard deviation (SD) rhinoceroses’ weight was 1311 ± 115 kg.

| Rhinoceros body mass range (kg) | Etorphine (mg)^a^ | Azaperone (mg)^b^ | Midazolam (mg)^c^ | Medetomidine (mg)^d^ | Butorphanol (mg)^e^ | Naltrexone (mg)^f^ |
| --- | --- | --- | --- | --- | --- | --- |
| 750 – 1000 | 2.00 | 10.0 | 10.0 | 5.00 | 20.0 | 40.0 |
| 1000 – 1250 | 2.50 | 12.5 | 12.5 | 6.25 | 25.0 | 50.0 |
| 1250 – 1500 | 3.00 | 15.0 | 15.0 | 7.50 | 30.0 | 60.0 |

^a^Etorphine (~0.002 mg/kg) ^b^Azaperone (~0.01 mg/kg ) ^c^Midazolam (~0.01 mg/kg) ^d^Medetomidine (~0.005 mg/kg) ^e^Butorphanol (10$\times$etorphine dose) ^f^Naltrexone (20$\times$etorphine dose).

Table S2 Sensitivity, specificity, positive and negative predictive values and area under the curve (AUC) values as a measure of clinical performance of the Nonin PalmSAT 2500A pulse oximeters’ and Masimo Radical-7 pulse co-oximeters at two measurement sites (third-eyelid and ear) for the detection of peripheral arterial oxygen-haemoglobin saturation (SpO_2_) ≤80% (31) in eight immobilised white rhinoceros (*Ceratotherium simum*). At SpO_2_ ≤80%, which indicates severe hypoxaemia, all the devices at the two measurement sites had low sensitivities and positive predictive values but high specificities and negative predictive values. As stated in the main document, in order to accurately detect hypoxaemia (or low blood oxygenation) in immobilised rhinoceroses with high risk of developing opioid-induced hypoxaemia, it is better to correctly identify hypoxeamia (i.e., high sensitivities) and miss normoxia (i.e., low specificities) so that interventions can be made to prevent tissue hypoxia, morbidity and mortality.

| **Device measurement site** | **Cut-off value (i.e., clinical decision limit)** | **Sensitivity (95% CI) (%)** | **Specificity (95% CI) (%)** | **Positive predictive value (95% CI) (%)** | **Negative predictive value (95% CI) (%)** | **AUC* ± 95% CI** | **P value** |
| --- | --- | --- | --- | --- | --- | --- | --- |
| Nonin third-eyelid | SpO_2_ ≤80% | 86 (76 to 93) | 95 (90 to 97) | 85 (73 to 91) | 95 (92 to 98) | 0.97 **±** 0.03^§§^ | <0.0001 |
| Masimo third-eyelid |  | 74 (69 to 84) | 93 (89 to 96) | 73 (59 to 83) | 94 (89 to 96) | 0.92 ± 0.04^§§^ | <0.0001 |
| Nonin ear |  | 76 (60 to 87) | 94 (90 to 97) | 71 (55 to 84) | 95 (91 to 98) | 0.92 ± 0.05^††^ | <0.0001 |
| Masimo ear |  | 70 (54 to 83) | 82 (76 to 87) | 46 (34 to 59) | 93 (87 to 96) | 0.85 ± 0.06^††^ | <0.0001 |
|  |  |  |  |  |  |  |  |

^*^ Area under the ROC curves (AUC) were used to determine the Nonin and Masimo device’s clinical performance in detecting clinical and severe hypoxaemia and the following guidelines have been proposed: low 0.50 < AUC ≤ 0.70**, moderate 0.70 <AUC ≤ 0.90^††^ and high 0.90 < AUC ≤ 1.00^§§^ clinical performance (30).

Table S3 Receiver operating characteristics (ROC) curves results. The yellow highlighted rows are the best cut-off values selected from the ROC curves (i.e., at this cut-off value, the devices were sensitive enough to detect hypoxaemia without compromising the specificity).

Nonin third-eyelid

| Cut-off values (%) | Sensitivity (%) | 95% confidence intervals | Specificity (%) | 95% confidence intervals | Likelihood ratio |
| --- | --- | --- | --- | --- | --- |
| < 35.00 | 1.136 | 0.2019% to 4.048% | 100.0 | 95.58% to 100.0% |  |
| < 37.50 | 1.705 | 0.4646% to 4.891% | 100.0 | 95.58% to 100.0% |  |
| < 39.50 | 2.841 | 1.219% to 6.477% | 100.0 | 95.58% to 100.0% |  |
| < 40.50 | 3.977 | 1.940% to 7.981% | 100.0 | 95.58% to 100.0% |  |
| < 41.50 | 4.545 | 2.321% to 8.712% | 100.0 | 95.58% to 100.0% |  |
| < 43.50 | 5.114 | 2.713% to 9.432% | 100.0 | 95.58% to 100.0% |  |
| < 45.50 | 5.682 | 3.115% to 10.14% | 100.0 | 95.58% to 100.0% |  |
| < 46.50 | 7.386 | 4.367% to 12.23% | 100.0 | 95.58% to 100.0% |  |
| < 47.50 | 7.955 | 4.797% to 12.91% | 100.0 | 95.58% to 100.0% |  |
| < 48.50 | 8.523 | 5.233% to 13.58% | 100.0 | 95.58% to 100.0% |  |
| < 50.00 | 9.091 | 5.673% to 14.26% | 100.0 | 95.58% to 100.0% |  |
| < 51.50 | 10.23 | 6.568% to 15.59% | 100.0 | 95.58% to 100.0% |  |
| < 52.50 | 10.80 | 7.021% to 16.24% | 100.0 | 95.58% to 100.0% |  |
| < 54.00 | 11.93 | 7.938% to 17.55% | 100.0 | 95.58% to 100.0% |  |
| < 55.50 | 12.50 | 8.402% to 18.20% | 100.0 | 95.58% to 100.0% |  |
| < 56.50 | 13.07 | 8.868% to 18.85% | 100.0 | 95.58% to 100.0% |  |
| < 57.50 | 13.64 | 9.338% to 19.49% | 100.0 | 95.58% to 100.0% |  |
| < 59.00 | 14.20 | 9.810% to 20.13% | 100.0 | 95.58% to 100.0% |  |
| < 60.50 | 14.77 | 10.29% to 20.77% | 100.0 | 95.58% to 100.0% |  |
| < 61.50 | 15.34 | 10.76% to 21.40% | 100.0 | 95.58% to 100.0% |  |
| < 62.50 | 15.91 | 11.24% to 22.03% | 100.0 | 95.58% to 100.0% |  |
| < 63.50 | 17.05 | 12.21% to 23.29% | 100.0 | 95.58% to 100.0% |  |
| < 65.00 | 18.75 | 13.67% to 25.16% | 100.0 | 95.58% to 100.0% |  |
| < 66.50 | 19.89 | 14.66% to 26.40% | 100.0 | 95.58% to 100.0% |  |
| < 67.50 | 20.45 | 15.16% to 27.01% | 100.0 | 95.58% to 100.0% |  |
| < 69.00 | 21.02 | 15.65% to 27.63% | 100.0 | 95.58% to 100.0% |  |
| < 71.00 | 22.16 | 16.65% to 28.85% | 100.0 | 95.58% to 100.0% |  |
| < 72.50 | 23.30 | 17.66% to 30.07% | 98.80 | 93.49% to 99.94% | 19.34 |
| < 74.00 | 25.00 | 19.18% to 31.89% | 98.80 | 93.49% to 99.94% | 20.75 |
| < 75.50 | 26.14 | 20.20% to 33.09% | 98.80 | 93.49% to 99.94% | 21.69 |
| < 76.50 | 26.70 | 20.72% to 33.69% | 98.80 | 93.49% to 99.94% | 22.16 |
| < 77.50 | 27.84 | 21.75% to 34.88% | 98.80 | 93.49% to 99.94% | 23.11 |
| < 78.50 | 29.55 | 23.30% to 36.66% | 98.80 | 93.49% to 99.94% | 24.52 |
| < 79.50 | 31.82 | 25.39% to 39.02% | 98.80 | 93.49% to 99.94% | 26.41 |
| < 80.50 | 35.23 | 28.55% to 42.53% | 98.80 | 93.49% to 99.94% | 29.24 |
| < 81.50 | 39.20 | 32.30% to 46.57% | 98.80 | 93.49% to 99.94% | 32.54 |
| < 82.50 | 46.02 | 38.82% to 53.39% | 97.59 | 91.63% to 99.57% | 19.10 |
| < 83.50 | 50.00 | 42.69% to 57.31% | 97.59 | 91.63% to 99.57% | 20.75 |
| < 84.50 | 57.95 | 50.57% to 65.00% | 97.59 | 91.63% to 99.57% | 24.05 |
| < 85.50 | 64.20 | 56.89% to 70.91% | 97.59 | 91.63% to 99.57% | 26.64 |
| < 86.50 | 66.48 | 59.22% to 73.03% | 97.59 | 91.63% to 99.57% | 27.59 |
| < 87.50 | 71.59 | 64.52% to 77.74% | 95.18 | 88.25% to 98.11% | 14.86 |
| < 88.50 | 78.98 | 72.37% to 84.35% | 95.18 | 88.25% to 98.11% | 16.39 |
| < 89.50 | 80.68 | 74.22% to 85.83% | 89.16 | 80.66% to 94.19% | 7.441 |
| < 90.50 | 85.23 | 79.23% to 89.71% | 83.13 | 73.66% to 89.68% | 5.053 |
| < 91.50 | 90.34 | 85.08% to 93.88% | 75.90 | 65.69% to 83.83% | 3.749 |
| < 92.50 | 93.75 | 89.16% to 96.47% | 68.67 | 58.06% to 77.64% | 2.993 |
| < 93.50 | 97.16 | 93.52% to 98.78% | 59.04 | 48.29% to 68.99% | 2.372 |
| < 94.50 | 97.73 | 94.30% to 99.11% | 46.99 | 36.62% to 57.62% | 1.843 |
| < 95.50 | 99.43 | 96.85% to 99.97% | 36.14 | 26.63% to 46.88% | 1.557 |
| < 96.50 | 99.43 | 96.85% to 99.97% | 28.92 | 20.27% to 39.43% | 1.399 |
| < 97.50 | 100.0 | 97.86% to 100.0% | 21.69 | 14.18% to 31.70% | 1.277 |
| < 98.50 | 100.0 | 97.86% to 100.0% | 4.819 | 1.890% to 11.75% | 1.051 |
| < 99.50 | 100.0 | 97.86% to 100.0% | 2.410 | 0.4281% to 8.366% | 1.025 |

Masimo third eyelid

| Cut-off values (%) | Sensitivity (%) | 95% confidence intervals | Specificity (%) | 95% confidence intervals | Likelihood ratio |
| --- | --- | --- | --- | --- | --- |
| < 21.00 | 0.4878 | 0.02502% to 2.711% | 100.0 | 95.77% to 100.0% |  |
| < 25.00 | 0.9756 | 0.1733% to 3.487% | 100.0 | 95.77% to 100.0% |  |
| < 27.50 | 1.951 | 0.7613% to 4.909% | 100.0 | 95.77% to 100.0% |  |
| < 28.50 | 2.439 | 1.046% to 5.582% | 100.0 | 95.77% to 100.0% |  |
| < 29.50 | 2.927 | 1.348% to 6.237% | 100.0 | 95.77% to 100.0% |  |
| < 30.50 | 3.902 | 1.990% to 7.510% | 100.0 | 95.77% to 100.0% |  |
| < 31.50 | 4.390 | 2.327% to 8.132% | 100.0 | 95.77% to 100.0% |  |
| < 33.00 | 4.878 | 2.671% to 8.745% | 100.0 | 95.77% to 100.0% |  |
| < 36.00 | 5.366 | 3.022% to 9.351% | 100.0 | 95.77% to 100.0% |  |
| < 39.00 | 6.341 | 3.743% to 10.55% | 100.0 | 95.77% to 100.0% |  |
| < 41.00 | 6.829 | 4.111% to 11.14% | 100.0 | 95.77% to 100.0% |  |
| < 44.00 | 7.317 | 4.484% to 11.72% | 98.85 | 93.77% to 99.94% | 6.366 |
| < 46.50 | 9.268 | 6.014% to 14.02% | 98.85 | 93.77% to 99.94% | 8.063 |
| < 47.50 | 9.756 | 6.405% to 14.59% | 98.85 | 93.77% to 99.94% | 8.488 |
| < 48.50 | 10.24 | 6.798% to 15.15% | 98.85 | 93.77% to 99.94% | 8.912 |
| < 50.00 | 10.73 | 7.195% to 15.71% | 98.85 | 93.77% to 99.94% | 9.337 |
| < 51.50 | 11.22 | 7.593% to 16.27% | 98.85 | 93.77% to 99.94% | 9.761 |
| < 52.50 | 12.68 | 8.804% to 17.93% | 98.85 | 93.77% to 99.94% | 11.03 |
| < 54.50 | 13.17 | 9.212% to 18.48% | 98.85 | 93.77% to 99.94% | 11.46 |
| < 57.00 | 13.66 | 9.622% to 19.03% | 98.85 | 93.77% to 99.94% | 11.88 |
| < 59.50 | 14.63 | 10.45% to 20.12% | 98.85 | 93.77% to 99.94% | 12.73 |
| < 61.50 | 15.12 | 10.86% to 20.66% | 98.85 | 93.77% to 99.94% | 13.16 |
| < 62.50 | 15.61 | 11.28% to 21.21% | 98.85 | 93.77% to 99.94% | 13.58 |
| < 63.50 | 18.05 | 13.39% to 23.89% | 98.85 | 93.77% to 99.94% | 15.70 |
| < 65.00 | 18.54 | 13.81% to 24.42% | 98.85 | 93.77% to 99.94% | 16.13 |
| < 66.50 | 19.02 | 14.24% to 24.95% | 98.85 | 93.77% to 99.94% | 16.55 |
| < 67.50 | 19.51 | 14.67% to 25.48% | 98.85 | 93.77% to 99.94% | 16.98 |
| < 69.00 | 20.49 | 15.53% to 26.53% | 98.85 | 93.77% to 99.94% | 17.82 |
| < 70.50 | 21.46 | 16.40% to 27.58% | 98.85 | 93.77% to 99.94% | 18.67 |
| < 71.50 | 21.95 | 16.83% to 28.10% | 98.85 | 93.77% to 99.94% | 19.10 |
| < 72.50 | 22.93 | 17.70% to 29.15% | 97.70 | 92.00% to 99.59% | 9.973 |
| < 73.50 | 23.90 | 18.58% to 30.19% | 97.70 | 92.00% to 99.59% | 10.40 |
| < 74.50 | 25.37 | 19.90% to 31.74% | 97.70 | 92.00% to 99.59% | 11.03 |
| < 75.50 | 26.83 | 21.23% to 33.28% | 97.70 | 92.00% to 99.59% | 11.67 |
| < 76.50 | 28.29 | 22.57% to 34.81% | 97.70 | 92.00% to 99.59% | 12.31 |
| < 77.50 | 30.24 | 24.37% to 36.85% | 97.70 | 92.00% to 99.59% | 13.16 |
| < 78.50 | 32.68 | 26.63% to 39.37% | 97.70 | 92.00% to 99.59% | 14.22 |
| < 79.50 | 37.07 | 30.76% to 43.87% | 95.40 | 88.77% to 98.20% | 8.063 |
| < 80.50 | 38.54 | 32.14% to 45.35% | 95.40 | 88.77% to 98.20% | 8.382 |
| < 81.50 | 41.46 | 34.94% to 48.30% | 94.25 | 87.24% to 97.52% | 7.215 |
| < 82.50 | 44.88 | 38.23% to 51.72% | 94.25 | 87.24% to 97.52% | 7.809 |
| < 83.50 | 49.76 | 42.98% to 56.54% | 93.10 | 85.76% to 96.80% | 7.215 |
| < 84.50 | 55.12 | 48.28% to 61.77% | 93.10 | 85.76% to 96.80% | 7.993 |
| < 85.50 | 62.93 | 56.13% to 69.24% | 93.10 | 85.76% to 96.80% | 9.124 |
| < 86.50 | 68.29 | 61.64% to 74.28% | 91.95 | 84.31% to 96.05% | 8.488 |
| < 87.50 | 71.22 | 64.68% to 76.98% | 89.66 | 81.50% to 94.46% | 6.885 |
| < 88.50 | 80.98 | 75.05% to 85.76% | 88.51 | 80.12% to 93.64% | 7.045 |
| < 89.50 | 84.39 | 78.79% to 88.72% | 85.06 | 76.10% to 91.05% | 5.648 |
| < 90.50 | 90.73 | 85.98% to 93.99% | 83.91 | 74.78% to 90.17% | 5.638 |
| < 91.50 | 92.20 | 87.70% to 95.14% | 72.41 | 62.23% to 80.71% | 3.342 |
| < 92.50 | 94.15 | 90.05% to 96.62% | 65.52 | 55.06% to 74.66% | 2.730 |
| < 93.50 | 97.07 | 93.76% to 98.65% | 57.47 | 46.98% to 67.33% | 2.283 |
| < 94.50 | 98.54 | 95.79% to 99.60% | 47.13 | 36.98% to 57.51% | 1.864 |
| < 95.50 | 98.54 | 95.79% to 99.60% | 35.63 | 26.37% to 46.11% | 1.531 |
| < 96.50 | 99.02 | 96.51% to 99.83% | 28.74 | 20.29% to 38.98% | 1.390 |
| < 97.50 | 99.02 | 96.51% to 99.83% | 11.49 | 6.365% to 19.88% | 1.119 |
| < 98.50 | 99.51 | 97.29% to 99.97% | 5.747 | 2.480% to 12.76% | 1.056 |

Nonin ear

| Cut-off value (%) | Sensitivity (%) | 95% confidence intervals | Specificity (%) | 95% confidence intervals | Likelihood ratio |
| --- | --- | --- | --- | --- | --- |
| < 19.00 | 0.5814 | 0.02982% to 3.219% | 100.0 | 95.58% to 100.0% |  |
| < 22.50 | 1.163 | 0.2066% to 4.140% | 100.0 | 95.58% to 100.0% |  |
| < 26.00 | 1.744 | 0.4754% to 5.002% | 100.0 | 95.58% to 100.0% |  |
| < 36.00 | 2.326 | 0.9080% to 5.826% | 100.0 | 95.58% to 100.0% |  |
| < 45.00 | 2.907 | 1.248% to 6.624% | 100.0 | 95.58% to 100.0% |  |
| < 48.50 | 3.488 | 1.608% to 7.401% | 100.0 | 95.58% to 100.0% |  |
| < 51.50 | 4.070 | 1.985% to 8.161% | 100.0 | 95.58% to 100.0% |  |
| < 54.00 | 5.233 | 2.777% to 9.644% | 100.0 | 95.58% to 100.0% |  |
| < 55.50 | 6.977 | 4.036% to 11.80% | 100.0 | 95.58% to 100.0% |  |
| < 56.50 | 7.558 | 4.470% to 12.50% | 100.0 | 95.58% to 100.0% |  |
| < 57.50 | 8.140 | 4.910% to 13.20% | 100.0 | 95.58% to 100.0% |  |
| < 58.50 | 8.721 | 5.356% to 13.89% | 100.0 | 95.58% to 100.0% |  |
| < 59.50 | 9.884 | 6.263% to 15.26% | 100.0 | 95.58% to 100.0% |  |
| < 60.50 | 10.47 | 6.723% to 15.93% | 100.0 | 95.58% to 100.0% |  |
| < 61.50 | 11.05 | 7.187% to 16.61% | 100.0 | 95.58% to 100.0% |  |
| < 62.50 | 13.37 | 9.078% to 19.27% | 100.0 | 95.58% to 100.0% |  |
| < 63.50 | 14.53 | 10.04% to 20.58% | 100.0 | 95.58% to 100.0% |  |
| < 64.50 | 14.53 | 10.04% to 20.58% | 98.80 | 93.49% to 99.94% | 12.06 |
| < 65.50 | 16.28 | 11.51% to 22.52% | 98.80 | 93.49% to 99.94% | 13.51 |
| < 66.50 | 17.44 | 12.50% to 23.81% | 98.80 | 93.49% to 99.94% | 14.48 |
| < 67.50 | 18.60 | 13.50% to 25.08% | 98.80 | 93.49% to 99.94% | 15.44 |
| < 68.50 | 19.19 | 14.00% to 25.72% | 98.80 | 93.49% to 99.94% | 15.92 |
| < 69.50 | 20.93 | 15.52% to 27.61% | 98.80 | 93.49% to 99.94% | 17.37 |
| < 70.50 | 21.51 | 16.03% to 28.24% | 98.80 | 93.49% to 99.94% | 17.85 |
| < 71.50 | 23.26 | 17.57% to 30.11% | 98.80 | 93.49% to 99.94% | 19.30 |
| < 72.50 | 23.84 | 18.09% to 30.73% | 97.59 | 91.63% to 99.57% | 9.892 |
| < 73.50 | 25.00 | 19.12% to 31.97% | 96.39 | 89.90% to 99.01% | 6.917 |
| < 74.50 | 29.07 | 22.80% to 36.25% | 96.39 | 89.90% to 99.01% | 8.043 |
| < 75.50 | 30.81 | 24.40% to 38.07% | 96.39 | 89.90% to 99.01% | 8.525 |
| < 76.50 | 33.14 | 26.54% to 40.47% | 95.18 | 88.25% to 98.11% | 6.876 |
| < 77.50 | 33.72 | 27.08% to 41.07% | 95.18 | 88.25% to 98.11% | 6.997 |
| < 78.50 | 36.63 | 29.79% to 44.05% | 95.18 | 88.25% to 98.11% | 7.600 |
| < 79.50 | 37.79 | 30.89% to 45.23% | 95.18 | 88.25% to 98.11% | 7.842 |
| < 80.50 | 39.53 | 32.53% to 46.99% | 93.98 | 86.66% to 97.40% | 6.563 |
| < 81.50 | 41.86 | 34.74% to 49.33% | 93.98 | 86.66% to 97.40% | 6.949 |
| < 82.50 | 45.93 | 38.65% to 53.39% | 93.98 | 86.66% to 97.40% | 7.624 |
| < 83.50 | 49.42 | 42.04% to 56.82% | 92.77 | 85.11% to 96.64% | 6.836 |
| < 84.50 | 52.33 | 44.89% to 59.66% | 92.77 | 85.11% to 96.64% | 7.238 |
| < 85.50 | 58.72 | 51.25% to 65.81% | 91.57 | 83.60% to 95.85% | 6.963 |
| < 86.50 | 63.37 | 55.95% to 70.21% | 89.16 | 80.66% to 94.19% | 5.844 |
| < 87.50 | 67.44 | 60.12% to 74.00% | 85.54 | 76.41% to 91.53% | 4.665 |
| < 88.50 | 72.67 | 65.57% to 78.78% | 80.72 | 70.96% to 87.77% | 3.770 |
| < 89.50 | 77.91 | 71.14% to 83.46% | 80.72 | 70.96% to 87.77% | 4.041 |
| < 90.50 | 83.14 | 76.83% to 88.00% | 77.11 | 66.99% to 84.83% | 3.632 |
| < 91.50 | 87.21 | 81.39% to 91.40% | 75.90 | 65.69% to 83.83% | 3.619 |
| < 92.50 | 89.53 | 84.07% to 93.28% | 71.08 | 60.57% to 79.73% | 3.096 |
| < 93.50 | 92.44 | 87.50% to 95.53% | 65.06 | 54.34% to 74.44% | 2.646 |
| < 94.50 | 93.02 | 88.20% to 95.96% | 59.04 | 48.29% to 68.99% | 2.271 |
| < 95.50 | 97.09 | 93.38% to 98.75% | 54.22 | 43.55% to 64.51% | 2.121 |
| < 96.50 | 97.67 | 94.17% to 99.09% | 45.78 | 35.49% to 56.45% | 1.802 |
| < 97.50 | 98.84 | 95.86% to 99.79% | 40.96 | 31.01% to 51.71% | 1.674 |
| < 98.50 | 99.42 | 96.78% to 99.97% | 26.51 | 18.20% to 36.89% | 1.353 |
| < 99.50 | 100.0 | 97.82% to 100.0% | 19.28 | 12.23% to 29.04% | 1.239 |

Masimo ear

| Cut-off values (%) | Sensitivity (%) | 95% confidence intervals | Specificity (%) | 95% confidence intervals | Likelihood ratio |
| --- | --- | --- | --- | --- | --- |
| < 17.50 | 0.5848 | 0.03000% to 3.238% | 100.0 | 95.42% to 100.0% |  |
| < 19.50 | 1.170 | 0.2078% to 4.164% | 100.0 | 95.42% to 100.0% |  |
| < 22.50 | 1.754 | 0.4782% to 5.030% | 100.0 | 95.42% to 100.0% |  |
| < 25.50 | 2.924 | 1.255% to 6.661% | 100.0 | 95.42% to 100.0% |  |
| < 28.00 | 3.509 | 1.618% to 7.443% | 100.0 | 95.42% to 100.0% |  |
| < 29.50 | 4.094 | 1.997% to 8.208% | 100.0 | 95.42% to 100.0% |  |
| < 31.00 | 4.678 | 2.389% to 8.959% | 100.0 | 95.42% to 100.0% |  |
| < 33.50 | 4.678 | 2.389% to 8.959% | 98.75 | 93.25% to 99.94% | 3.743 |
| < 39.00 | 5.848 | 3.207% to 10.43% | 98.75 | 93.25% to 99.94% | 4.678 |
| < 43.50 | 6.433 | 3.630% to 11.15% | 98.75 | 93.25% to 99.94% | 5.146 |
| < 45.00 | 7.018 | 4.060% to 11.86% | 98.75 | 93.25% to 99.94% | 5.614 |
| < 47.00 | 7.602 | 4.496% to 12.57% | 98.75 | 93.25% to 99.94% | 6.082 |
| < 50.00 | 8.187 | 4.939% to 13.27% | 98.75 | 93.25% to 99.94% | 6.550 |
| < 53.00 | 8.772 | 5.388% to 13.97% | 98.75 | 93.25% to 99.94% | 7.018 |
| < 54.50 | 9.357 | 5.842% to 14.66% | 98.75 | 93.25% to 99.94% | 7.485 |
| < 55.50 | 9.942 | 6.300% to 15.34% | 98.75 | 93.25% to 99.94% | 7.953 |
| < 56.50 | 10.53 | 6.763% to 16.02% | 98.75 | 93.25% to 99.94% | 8.421 |
| < 57.50 | 11.11 | 7.230% to 16.70% | 98.75 | 93.25% to 99.94% | 8.889 |
| < 59.00 | 12.28 | 8.174% to 18.04% | 98.75 | 93.25% to 99.94% | 9.825 |
| < 60.50 | 12.87 | 8.652% to 18.71% | 98.75 | 93.25% to 99.94% | 10.29 |
| < 61.50 | 13.45 | 9.133% to 19.37% | 98.75 | 93.25% to 99.94% | 10.76 |
| < 63.00 | 14.04 | 9.616% to 20.03% | 98.75 | 93.25% to 99.94% | 11.23 |
| < 64.50 | 14.62 | 10.10% to 20.69% | 98.75 | 93.25% to 99.94% | 11.70 |
| < 66.00 | 15.79 | 11.08% to 22.00% | 98.75 | 93.25% to 99.94% | 12.63 |
| < 67.50 | 16.96 | 12.08% to 23.29% | 98.75 | 93.25% to 99.94% | 13.57 |
| < 68.50 | 18.13 | 13.08% to 24.58% | 98.75 | 93.25% to 99.94% | 14.50 |
| < 69.50 | 20.47 | 15.10% to 27.13% | 98.75 | 93.25% to 99.94% | 16.37 |
| < 70.50 | 22.22 | 16.64% to 29.03% | 98.75 | 93.25% to 99.94% | 17.78 |
| < 71.50 | 23.39 | 17.67% to 30.28% | 98.75 | 93.25% to 99.94% | 18.71 |
| < 72.50 | 23.98 | 18.19% to 30.90% | 98.75 | 93.25% to 99.94% | 19.18 |
| < 73.50 | 25.73 | 19.76% to 32.77% | 97.50 | 91.34% to 99.56% | 10.29 |
| < 74.50 | 27.49 | 21.34% to 34.62% | 97.50 | 91.34% to 99.56% | 10.99 |
| < 75.50 | 30.41 | 24.01% to 37.67% | 97.50 | 91.34% to 99.56% | 12.16 |
| < 76.50 | 33.33 | 26.70% to 40.70% | 97.50 | 91.34% to 99.56% | 13.33 |
| < 77.50 | 35.67 | 28.88% to 43.09% | 93.75 | 86.19% to 97.30% | 5.708 |
| < 78.50 | 37.43 | 30.52% to 44.88% | 92.50 | 84.59% to 96.52% | 4.990 |
| < 79.50 | 39.77 | 32.73% to 47.25% | 90.00 | 81.49% to 94.85% | 3.977 |
| < 80.50 | 44.44 | 37.20% to 51.93% | 87.50 | 78.50% to 93.07% | 3.556 |
| < 81.50 | 47.95 | 40.59% to 55.40% | 86.25 | 77.03% to 92.15% | 3.488 |
| < 82.50 | 51.46 | 44.02% to 58.84% | 83.75 | 74.16% to 90.25% | 3.167 |
| < 83.50 | 53.80 | 46.33% to 61.11% | 83.75 | 74.16% to 90.25% | 3.311 |
| < 84.50 | 57.31 | 49.82% to 64.48% | 82.50 | 72.74% to 89.28% | 3.275 |
| < 85.50 | 60.82 | 53.34% to 67.82% | 80.00 | 69.95% to 87.30% | 3.041 |
| < 86.50 | 63.74 | 56.31% to 70.57% | 78.75 | 68.58% to 86.29% | 3.000 |
| < 87.50 | 68.42 | 61.11% to 74.92% | 75.00 | 64.52% to 83.19% | 2.737 |
| < 88.50 | 73.68 | 66.62% to 79.71% | 71.25 | 60.54% to 80.01% | 2.563 |
| < 89.50 | 80.12 | 73.50% to 85.41% | 67.50 | 56.64% to 76.76% | 2.465 |
| < 90.50 | 82.46 | 76.06% to 87.43% | 67.50 | 56.64% to 76.76% | 2.537 |
| < 91.50 | 86.55 | 80.63% to 90.87% | 62.50 | 51.55% to 72.31% | 2.308 |
| < 92.50 | 92.40 | 87.43% to 95.50% | 48.75 | 38.11% to 59.51% | 1.803 |
| < 93.50 | 95.91 | 91.79% to 98.00% | 41.25 | 31.11% to 52.20% | 1.632 |
| < 94.50 | 96.49 | 92.56% to 98.38% | 33.75 | 24.35% to 44.64% | 1.456 |
| < 95.50 | 97.08 | 93.34% to 98.74% | 23.75 | 15.76% to 34.14% | 1.273 |
| < 96.50 | 98.83 | 95.84% to 99.79% | 16.25 | 9.750% to 25.84% | 1.180 |
| < 97.50 | 100.0 | 97.80% to 100.0% | 8.750 | 4.303% to 16.98% | 1.096 |
| < 98.50 | 100.0 | 97.80% to 100.0% | 3.750 | 1.022% to 10.45% | 1.039 |
| < 99.50 | 100.0 | 97.80% to 100.0% | 2.500 | 0.4442% to 8.664% | 1.026 |
